# Prostate cancer-associated transcript 6 (PCAT6) promotes epithelial-mesenchymal transition and stemness and worsens prognosis in patients with colorectal cancer: PCAT6 promotes CRC metastasis

**DOI:** 10.3724/abbs.2024031

**Published:** 2024-04-11

**Authors:** Xun Sun, Yitao Yuan, Suyao Li, Lu Gan, Midie Xu, Qingguo Li, Mengling Liu, Keshu Hu, Ke Nan, Jiayu Zhang, Yu Dong, Yufu Lin, Xiuping Zhang, Pengcong Hou, Tianshu Liu

**Affiliations:** 1 Department of Medical Oncology Zhongshan Hospital Fudan University Shanghai 200032 China; 2 Cancer Center Zhongshan Hospital Fudan University Shanghai 200032 China; 3 Shanghai Institute of Precision Medicine Shanghai Ninth People’s Hospital Shanghai Jiao Tong University School of Medicine Shanghai 200032 China; 4 Department of Anesthesiology Zhongshan Hospital Fudan University Shanghai 200032 China; 5 Fudan Zhangjiang Institute Shanghai 201203 China; 6 Department of Pathology and Tissue Bank Fudan University Shanghai Cancer Center Shanghai 200032 China; 7 Department of Colorectal Surgery Fudan University Shanghai Cancer Center Shanghai 200032 China; 8 Department of Oncology Shanghai Medical College Fudan University Shanghai 200032 China; 9 Department of Oncology Zhongshan Hospital (Xiamen) Fudan University Xiamen 361015 China; 10 Xiamen Clinical Research Center for Cancer Therapy Xiamen Branch Zhongshan Hospital Fudan University Xiamen 361015 China

**Keywords:** colorectal cancer, lncRNA, PCAT6, metastasis

## Abstract

Approximately 20% of colorectal cancer (CRC) patients are first diagnosed with metastatic colorectal cancer (mCRC) because they develop symptoms at an advanced stage. Despite advancements in treatment, patients with metastatic disease still experience inferior survival rates. Our objective is to investigate the association between long noncoding RNAs (lncRNAs) and prognosis and to explore their role in mCRC. In this study, we find that elevated expression of PCAT6 is independently linked to unfavourable survival outcomes in The Cancer Genome Atlas (TCGA) data, and this finding is further confirmed in CRC samples obtained from Fudan University Shanghai Cancer Center. Cell lines and xenograft mouse models are used to examine the impact of PCAT6 on tumor metastasis. Knockdown of *PCAT6* is observed to impede the metastatic phenotype of CRC, as evidenced by functional assays, demonstrating the suppression of epithelial-mesenchymal transition (EMT) and stemness. Our findings show the significance of PCAT6 in mCRC and its potential use as a prognostic biomarker.

## Introduction

Although early screening, diagnosis, and treatment of colorectal cancer (CRC) have advanced over the decades, approximately 20% of CRC patients suffer from distant metastases at the time of initial diagnosis, whereas up to 50% of patients with initially localized disease will develop metastases [1]. Due to distant metastasis, CRC is one of the leading causes of cancer-associated death. The liver and lung are the two most common metastatic sites of CRC. Approximately 50%‒60% of patients with orthotopic CRC develop metastases, 80%‒90% of which are unresectable liver metastases [2]. Approximately 10%‒25% of patients with CRC *in situ* develop lung metastases [3]. The 5-year survival rate of patients with early-stage CRC is greater than 90%, while that of patients with advanced CRC remains low, at <14% [4]. Hence, it is crucial to investigate the mechanisms linked to mCRC to develop innovative therapeutic approaches targeting mCRC.

The epithelial-mesenchymal transition (EMT) is a regulatory factor in the process of metastasis. EMT properties, such as cytoskeletal deformability and motility, are accompanied by the presence of EMT markers, including E-cadherin, N-cadherin and Snail [5]. In head and neck cancers, cells exhibiting EMT properties also exhibit increased expressions of cancer stem cell (CSC) markers such as CD44, CD133 and SOX2. This observation establishes a positive association between EMT and stemness [ 6, 7]. Cancer stem cells, a distinct subset of cancer cells, have been implicated in cancer initiation and relapse. CD44, a biomarker of cancer stem cells, is associated with an unfavourable prognosis [8]. Similarly, CD133 has been identified as a prognostic marker for poor outcomes in patients with breast cancer [9].

PCAT6, also called KDM5B-AS1, is a member of the PCAT family. The *PCAT6* gene is located on chromosome 1q32.1 and has two expression regions. PCAT6 is an intergenic lncRNA. RNA-seq data from the Human Protein Atlas database showed that among normal tissues, human testicular tissue has the highest expression level of PCAT6. Compared with that in normal tissues, the expression of PCAT6 in tumor tissues is abnormally elevated. PCAT6 has been reported to be upregulated in many types of cancers including bladder cancer [10], breast cancer [11], cervical cancer [12], CRC [13], gastrointestinal stromal tumor [14], gastric cancer [15] and other cancers. In CRC, PCAT6 can promote apoptosis and the resistance of CRC cells to 5-FU [16]. These reports indicated that PCAT6 may serve as a potential therapeutic target for patients who are resistant to 5-FU. Although there is a dearth of comprehensive information regarding the prognosis, molecular mechanisms, and biological aspects of PCAT6, these findings have validated our initial hypothesis and have motivated us to pursue further investigation.

In the present study, we initially screened upregulated lncRNAs in metastatic colorectal cancer (mCRC) tissues. Subsequently, we correlated these lncRNAs with clinicopathological parameters, employing a screening criterion of a *P* value less than 0.05. Based on hazard ratio analysis, we identified that PCAT6 is significantly associated with patient prognosis.

## Materials and Methods

### Public database resources

Using The Cancer Genome Atlas (TCGA) biolinks package, basic clinical information of patients was obtained from the TCGA-COAD and TCGA-READ projects. The expression profiles of the tumor tissues were obtained from the TCGA ( https://www.cancer.gov/tcga).

Basic clinical information obtained from TCGA was organized via the tableone package, and the original gene expression data of CRC from TCGA were organized using human transcriptome gene annotation files from the GENCODE website ( www.genecodegenes.org). The DESeq2 package was used to standardize gene expression data and analyze differentially expressed genes (DEGs) by taking cancer status, adjacent cancer status, and TNM stage as grouping factors. Moreover, significantly highly expressed lncRNAs in cancer tissues and at stages III+IV were screened using the criteria of an absolute value of |log _2_ fold change (FC)|>1 and a corrected *P* value≤0.05. Ggplot2 was used to construct volcano plots of DEGs. The identified lncRNAs were then matched to the corresponding clinicopathological parameters, and proportional hazards hypothesis testing was performed using the survival package in R. Moreover, Cox regression analysis was performed, and the ggplot2 and VennDiagram packages were used to construct Venn diagrams and identify lncRNAs that are significantly associated with patient prognosis, using *P*<0.05 as the screening criterion. The top 10 lncRNAs were obtained in ascending order by hazard ratio (HR).

### Patients and clinical data collection

A total of 202 CRC patients diagnosed between March 2008 and November 2009 were included in this study. The inclusion criteria were as follows: (1) histologically confirmed colorectal adenocarcinoma and (2) underwent surgery for the primary colorectal tumor and received no systemic or local antitumor therapy before surgery. The exclusion criteria were as follows: (1) incomplete colorectal cancer primary tumor tissue samples and (2) patients with other primary malignant tumors or with a family history of hereditary tumors. The basic clinical and pathological characteristics included sex, age, primary tumor site, node invasion status, metastatic state and postoperative pathological stage. TNM stage was evaluated according to the 8th edition of the AJCC on Cancer Staging Manual [17]. Overall survival (OS) was recorded as the time from surgery to death or the last follow-up (September 2015). This study was approved by the Ethics Committee of Fudan University Shanghai Cancer Center (050432-4-2108*). Informed consent was obtained from all patients.

### Cell lines and reagents

Human CRC cell lines, including Caco2, SW620, HCT116, COLO205, HT29, SW480 and DLD-1, were purchased from the Chinese Academy of Sciences Cell Bank (Shanghai, China) and cultured in high-glucose DMEM (HyClone, Logan, USA) supplemented with 10% fetal bovine serum (Gibco, Carlsbad, USA) and 1% penicillin/streptomycin (Gibco). All cells were cultured at 37°C with 5% CO _2_.

### Construction of stable cell lines

The plasmids used for *PCAT6* silencing were purchased from Genomeditech (Shanghai, China). Lentiviruses were generated using HEK293 T cells and transduced into target cells. The stably transfected cells were selected using puromycin. The short hairpin RNA (shRNA) targeting sequences of PCAT6 were as follows: shPCAT6#2, 5′-GCCTTGCTCGTGCTTCTACCA-3′, shPCAT6#3, 5′-GAATGTTTGCTGTCAGATGTC-3′, and shCtrl, 5′- TTCTCCGAACGTGTCACGT-3′

### Reverse transcription-quantitative polymerase chain reaction (RT-qPCR)

Total RNA was extracted from cells using an EZ-press RNA Purification kit (B0004D; EZBioscience, Roseville, USA) according to the manufacturer’s instructions. RNA was reverse-transcribed using a PrimeScript RT Master Mix (Perfect Real Time) kit (RR036A; TaKaRa, Dalian, China). A TB Green Premix Ex Taq (Tli RNase H Plus) kit (RR420A; TaKaRa) was used for quantitative RT-qPCR. Primers for *PCAT6* and *β*- *actin* were synthesized by Tsingke Biotechnology (Beijing, China) with the following sequences: *β*- *actin*-F, 5′-ATTGATTCGAAACCTTGCCC-3′/ *β*- *actin*-R, 5′-AGCTCCAGTACACCCTTCTA-3′; and *PCAT6*-F, 5′-TCCTCATTCGGTCCATCCAACTCC-3′/ *PCAT6*-R, 5′-GAAGCACGAGCAAGGCAGAGAC-3′. The 2 ^‒∆∆Ct^ method was used for the relative quantitative analysis of expression data, using *β*- *actin* as an internal reference gene.

### Western blot analysis

Cells were lysed in RIPA buffer (Solarbio, Shanghai, China) supplemented with a proteinase/phosphatase inhibitor mixture (Beyotime Biotechnology, Shanghai, China) for protein extraction. A BCA kit (Beyotime Biotechnology) was used to determine the total protein concentration. Proteins were then separated on 10% sodium dodecylsulfate-polyacrylamide gels and transferred onto nitrocellulose membranes (Millipore, Billerica, USA). After being blocked with 5% skim milk, the membranes were incubated with diluted primary antibodies overnight at 4°C. The membranes were washed and incubated with horseradish peroxidase-conjugated secondary antibodies for 1 h at room temperature. The protein bands were visualized using enhanced chemiluminescence reagent. The antibodies used were as follows: epithelia-mesenchymal transition (EMT) antibody sample kit (#9782; CST, Beverly, USA), anti-β-actin antibody (#100166-mm10; Sino Biological, Shanghai, China), HRP-linked anti-rabbit IgG antibody (#7074; CST), and HRP-linked anti-mouse IgG antibody (#7076; CST). Semiquantitative analysis of the bands was performed using ImageJ software (version 1.53a).

### Transwell assay

For the transwell assay, 800 μL of DMEM supplemented with 20% FBS was added to the lower chamber, while 8×10 ^4^ HCT116 cells or 4×10 ^4^ SW480 cells in serum-free media were added to the upper chamber. After 48‒72 h, the cells were fixed with 4% paraformaldehyde at room temperature for 10‒20 min. The chambers were washed with phosphate-buffered saline (PBS) twice, stained with 1% crystal violet for 10 min, and washed twice with PBS. The remaining cells on the upper surface of the chamber were removed using a cotton-tipped swab, and the cells on the lower surface of the chamber were counted.

### Immunofluorescence staining

Immunofluorescence microscopy was used to detect the localization and expression of target proteins. First, the cells were fixed with 4% paraformaldehyde, incubated with 0.5% Triton X-100, and blocked with 5% FBS. The cells were then incubated with primary antibodies at 4°C overnight, followed by incubation with a fluorescent secondary antibody. The antibodies used were as follows: anti-E-cadherin antibody (#3195; CST), anti-N-cadherin antibody (#13116; CST), anti-rabbit IgG (H+L), and F(ab′)2 fragment (Alexa Fluor® 488 Conjugate) (#4412; CST). Cell nuclei were stained with 4,6-diamidino-2-phenylindole (DAPI). Images were obtained using a confocal laser scanning microscope (Nikon, Tokyo, Janpan).

### Sphere formation assay

CRC cells were plated in ultralow attachment 96-well plates (Corning, New York, USA) at a density of 80 cells per well and cultured in serum-free DMEM/F12 basal medium supplemented with B-27 supplement (1:50), 10 ng/mL human epidermal growth factor, and 10 ng/mL human fibroblast growth factor-2 at 37°C for 2 weeks. Thereafter, the cell sphere diameters were measured, and spheres with a diameter>100 μm were counted as primary spheres.

### Tail vein metastatic mouse model

HCT116-shCtrl and HCT116-shPCAT6#3 cells in the logarithmic growth phase were digested with trypsin and harvested. The cells were resuspended in PBS to a density of 2×10 ^6^ cells/100 μL and injected into nude mice via the tail vein. The activity and defecation of the mice were observed on the following day. After 6 weeks, the mice were sacrificed, and the number of tumors in the lungs and livers of the mice was counted. Tumors were fixed in 4% paraformaldehyde for H&E staining.

### H&E staining

Dehydrate the fixed tissue samples and embed them in paraffin, creating tissue sections of 4‒6 mm thickness. Wash the paraffin sections in xylene I for 5 min and xylene II for 5 min. Subsequently, wash the sections in absolute ethanol for 1 min. Immerse the sections in hematoxylin staining solution for 5‒30 s, adjusting the time according to the concentration of the hematoxylin solution. After staining, rinse the sections with running tap water for 3 times, each lasting 1 min. Place the sections in hydrochloric acid alcohol for 10 s, followed by rinsing with running water and counterstaining with 1% ammonia water for 1 min before rinsing with running water again. Immerse the sections in eosin staining solution for 1‒3 min. Then, dehydrate and clear the sections by sequentially placing them in 95% ethanol I for 5 min, 95% ethanol II for 5 min, absolute ethanol I for 5 min, absolute ethanol II for 5 min, xylene I for 5 min, and xylene II for 5 min. After dehydration and clearing, remove the sections from xylene and allow them to air-dry slightly. Seal the sections with a neutral mounting medium and observe them under a microscope for examination and photography.

### Statistical analysis

Differential gene expression, Kaplan-Meier survival, and univariate and multivariate Cox proportional hazards model analysis were performed using the Dseq2 package in R. In addition to the statistical test methods included in the analysis model, chi-square tests, Student’s *t* tests, Welch’s tests, Wilcoxon rank-sum tests, and log-rank tests were also used. Statistical analysis was performed using two-sided tests (if applicable), with *P*<0.05 as the standard of statistical significance. Software programs, such as RStudio, GraphPad Prism, and ImageJ, were used for data analysis and graph preparation.

## Results

### PCAT6 is upregulated in CRC tissues

**Figure 1:**
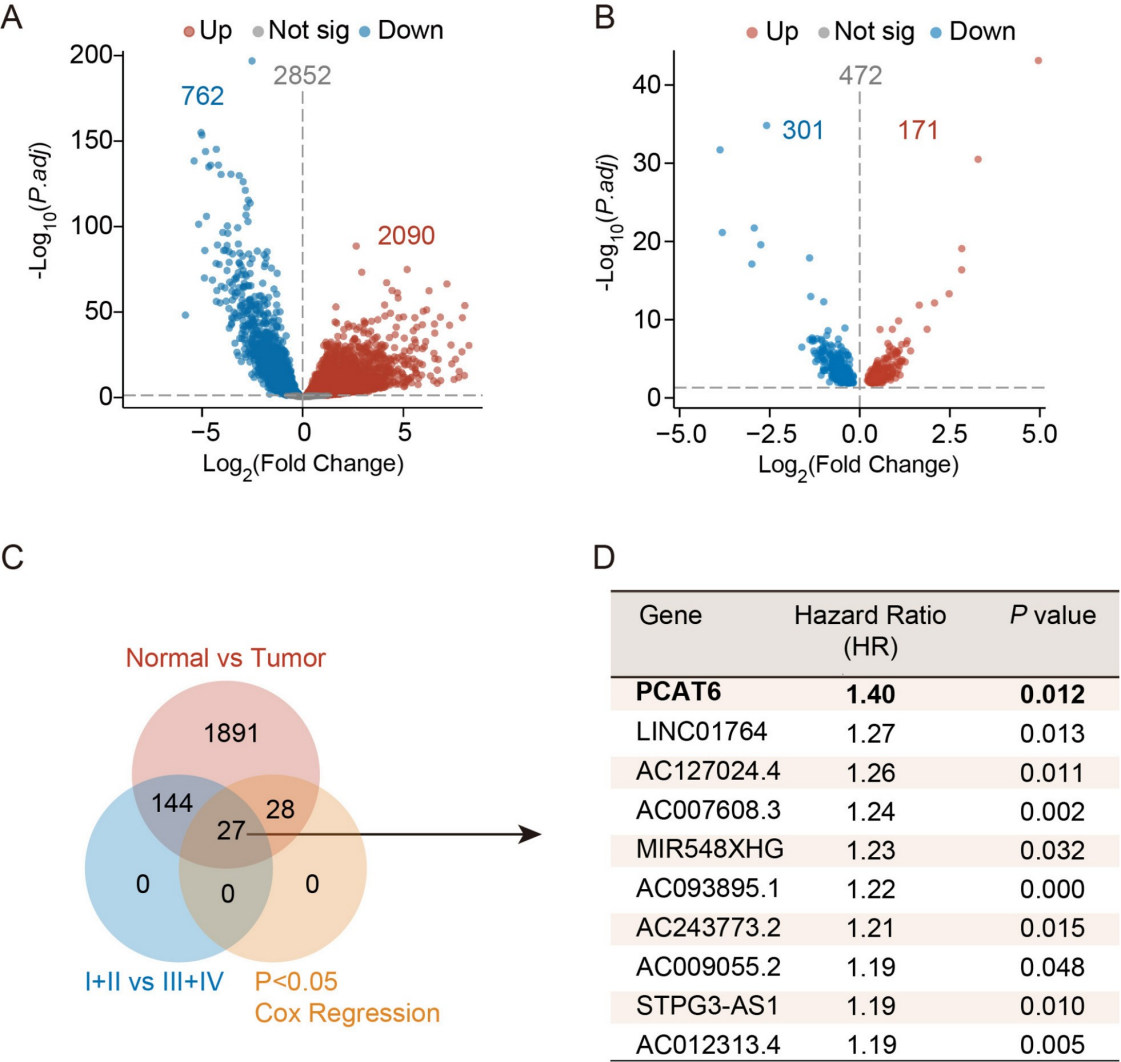
Figure 1 PCAT6 is upregulated in colorectal cancer tissues (A) Volcano plots showing the DEGs in colorectal cancer tissues and normal tissues in TCGA database. The cut-off was |log2FC|>1 and P value≤0.05. (B) Volcano plots showing the DEGs in stage III+IV CRC tissues compared with stage I+II CRC tissues in TCGA. (C) Venn diagram of upregulated lncRNAs, upregulated lncRNAs in III+IV CRC tissues, and lncRNAs correlated with prognosis. (D) P values and hazard ratios (HR) of the top 10 lncRNAs.

To identify lncRNAs that contribute to CRC development, we performed differential expression analysis using the TCGA dataset. In this dataset, a total of 2852 significantly differentially expressed lncRNAs in CRC tissues were identified, among which 2090 were upregulated, whereas 762 were downregulated ( Figure 1A). The TNM stage is a key factor in defining tumor progression. Thus, we used the TNM stage as the grouping criterion and identified 171 upregulated and 301 downregulated lncRNAs in stages III+IV ( Figure 1B). We then matched 171 lncRNAs with clinicopathological features, analyzed the relationship between lncRNAs and patient prognosis, and calculated the hazard ratio (HR). At this point, PCAT6 was selected based on *P*<0.05 and HR ( Figure 1C,D).

### PCAT6 indicates a poor prognosis for patients with CRC

Patients with metastatic CRC often have a poor prognosis. Therefore, we explored the relationship between PCAT6 expression and the prognosis of patients with CRC. We first analyzed the basic clinical characteristics of patients in the TCGA database. Basic information (sex and age) and expression profile data from 619 CRC patients were obtained from the TCGA database. The basic clinical characteristics of the patients are shown in Table 1. The patients were divided into high- and low-PCAT6 expression groups, with 310 and 309 patients, respectively. Cox regression analysis indicated that PCAT6 expression is significantly correlated with lymph node metastasis ( *P*=0.006), distant metastasis ( *P*=0.004), and TNM stage ( *P*=0.001) ( Table 1). This result suggested that PCAT6 may play an important regulatory role in the metastasis of CRC.

**Table:** **Table 1** Basic clinicopathological characteristics of patients in The Cancer Genome Atlas*

Characteristics	Subgroup	Number of cases (%)	PCAT6 expression	*P* value
Low ( *n*=309)	High ( *n*=310)
Sex	Female	289 (46.7)	151	138	0.278
	Male	330 (53.3)	158	172	
Age	≤ 65	269 (43.5)	133	136	0.835
	> 65	350 (56.5)	176	174	
T stage	T1	20 (3.2)	13	7	0.086
	T2	105 (17.1)	54	51	
	T3	422 (68.4)	214	208	
	T4	70 (11.3)	26	44	
N stage	N0	351 (57.0)	194	157	**0.006**
	N1	150 (24.4)	67	83	
	N2	115 (18.7)	46	69	
M stage	M0	459 (84.0)	247	212	**0.004**
	M1	87 (16.0)	32	55	
TNM stage	I	105 (17.5)	61	44	**0.001**
	II	227 (37.9)	127	100	
	III	179 (29.9)	80	99	
	IV	88 (14.7)	31	57	

*Partially missing patient data is not displayed.

**Figure 2:**
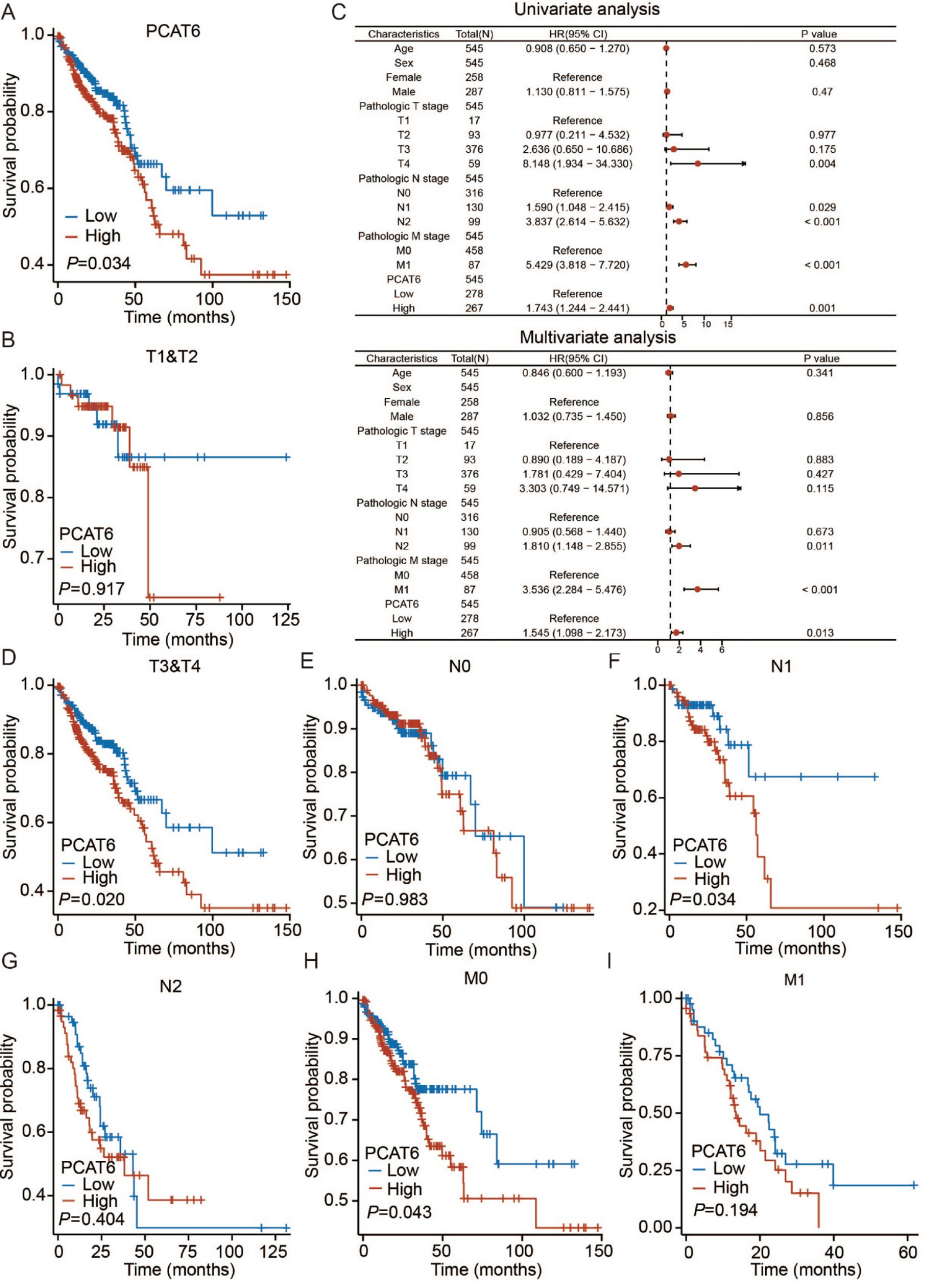
Figure 2 High PCAT6 expression is indicative of poor prognosis in patients with colorectal cancer in TCGA database (A) The correlation between PCAT6 expression and overall survival of patients. (C) The results of univariate and multivariate Cox regression analysis in the TCGA database. (B,D) The correlation between PCAT6 expression in the T stage subgroups and overall survival. (E‒G) The correlation between PCAT6 expression in the N stage subgroups and overall survival. (H,I) The correlation between PCAT6 expression in the M stage subgroups and overall survival.

Furthermore, patients with high PCAT6 expression had shorter OS ( *P*=0.034) ( Figure 2A). Moreover, after excluding missing values, the results of the univariate Cox regression model analysis indicated that the depth of invasion, lymph node metastasis, distant metastasis, and PCAT6 expression are prognostic factors in patients with CRC. We further performed Cox multivariate regression analysis to evaluate the prognostic significance of each factor and found that in addition to lymph node and distant metastasis, PCAT6 is an independent prognostic factor for CRC ( Figure 2C).

Based on the above analysis of basic clinicopathological characteristics, we found that PCAT6 expression is correlated with lymph node and distant metastasis. Therefore, we conducted a subgroup analysis of PCAT6 expression and prognosis. In the subgroup analysis of tumor invasion depth and T stage, we found that compared to patients with low PCAT6 expression in the T3+T4 stages, those with high PCAT6 expression had a significantly shorter OS, whereas no significant difference was observed in patients with T1+T2 stages ( Figure 2B,D). In terms of lymph node metastasis, in the N1 subgroup, patients with high PCAT6 expression had a significantly shorter OS than those with low PCAT6 expression, while no significant differences were observed between the N0 and N2 subgroups ( Figure 2E‒G). According to the distant metastasis subgroup analysis, in the M0 subgroup, patients with low PCAT6 expression had a significantly longer OS than those with high PCAT6 expression ( Figure 2H,I).

**Figure 3:**
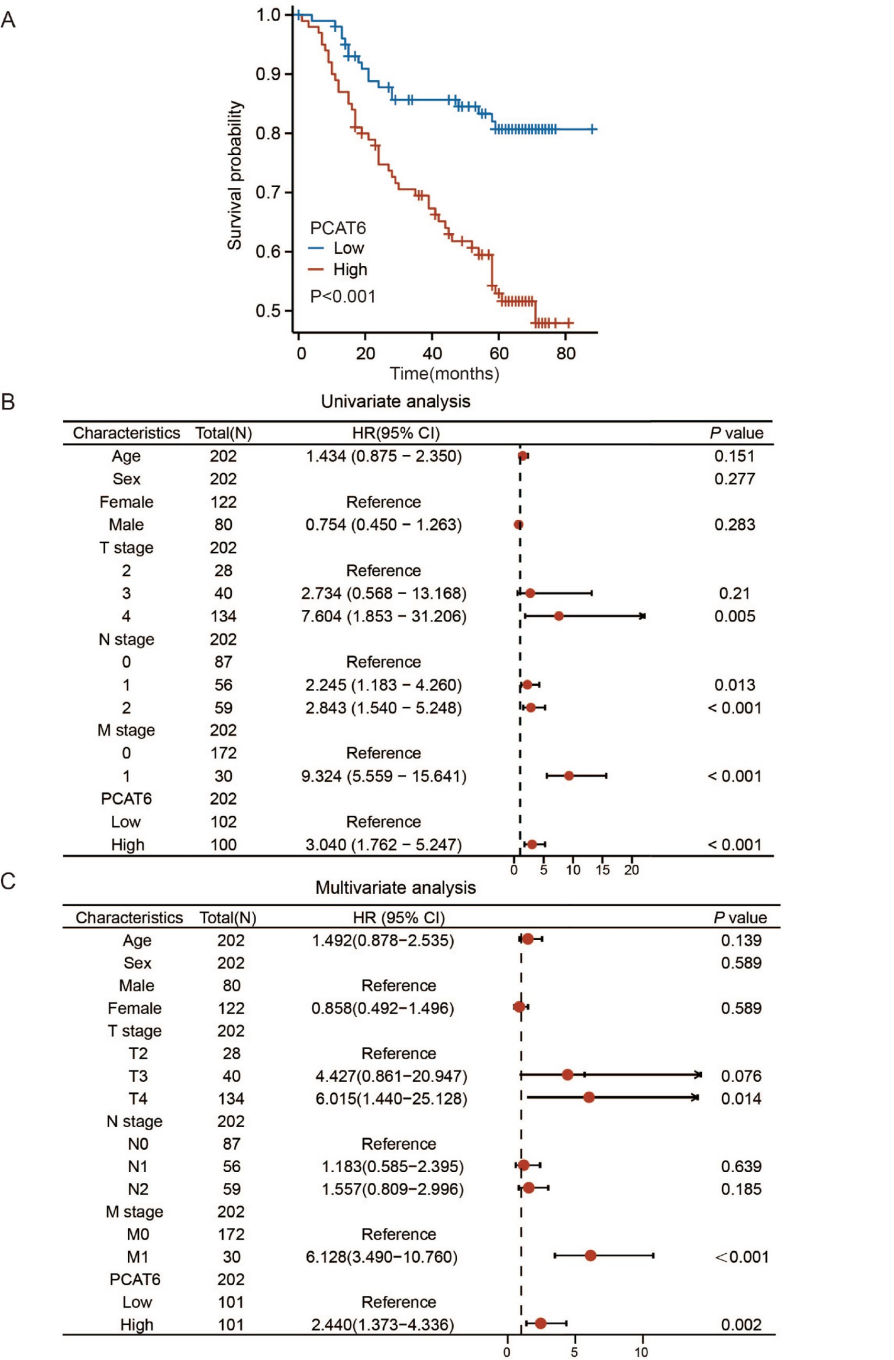
Figure 3 High PCAT6 level is associated with poor prognosis in patients with colorectal cancer (A) Overall survival of patients in the PCAT6-high group and PCAT6-low group. (B,C) The results of univariate and multivariate Cox regression analysis.

To further explore the prognostic value of PCAT6 in patients with CRC, we retrospectively collected data from patients who were first diagnosed with CRC at Fudan University Shanghai Cancer Center between 2008 and 2009 and who underwent radical resection of the primary tumor. A total of 202 patient tissue samples were collected and subjected to RT-qPCR to detect *PCAT6* expression. Among the 202 patients, 101 were designated as PCAT6-high, and 101 were designated as PCAT6-low. The basic clinicopathological characteristics of these patients are shown in Table 2. Kaplan-Meier analysis demonstrated that the survival time of patients with high PCAT6 expression was significantly shorter than that of patients with low PCAT6 expression ( Figure 3A). Single-factor Cox regression model analysis revealed that the depth of invasion, lymph node metastasis, distant metastasis, and PCAT6 expression are prognostic factors in patients with CRC. Cox multivariate regression analysis indicated that PCAT6 is an independent prognostic factor in patients with CRC ( Figure 3B,C). In summary, PCAT6 is an independent risk factor for poor prognosis in patients with CRC.

**Table:** **Table 2** Clinicopathological features of 202 CRC patients’ tissue samples

Characteristics	Subgroup	Number of cases (%)	PCAT6 expression	*P* value
Low ( *n*=101)	High ( *n*=101)
Sex	Female	122 (60.4)	59	63	0.565
	Male	80 (39.6)	42	38	
Age	< 60	121 (59.9)	61	60	0.886
	≥ 60	81 (40.1)	40	41	
T stage	T2	28 (13.9)	16	12	0.048
	T3	40 (19.8)	26	14	
	T4	134 (66.3)	59	75	
N stage	N0	87 (43.1)	50	37	0.165
	N1	56 (27.7)	26	30	
	N2	59 (29.2)	25	34	
M stage	M0	172 (85.1)	95	77	**<0.001**
	M1	30 (14.9)	6	24	
TNM stage	I	15 (7.4)	7	8	**0.001**
	II	59 (29.2)	38	21	
	III	98 (48.5)	50	48	
	IV	30 (14.9)	6	24	

### PCAT6 promotes CRC metastasis

**Figure 4:**
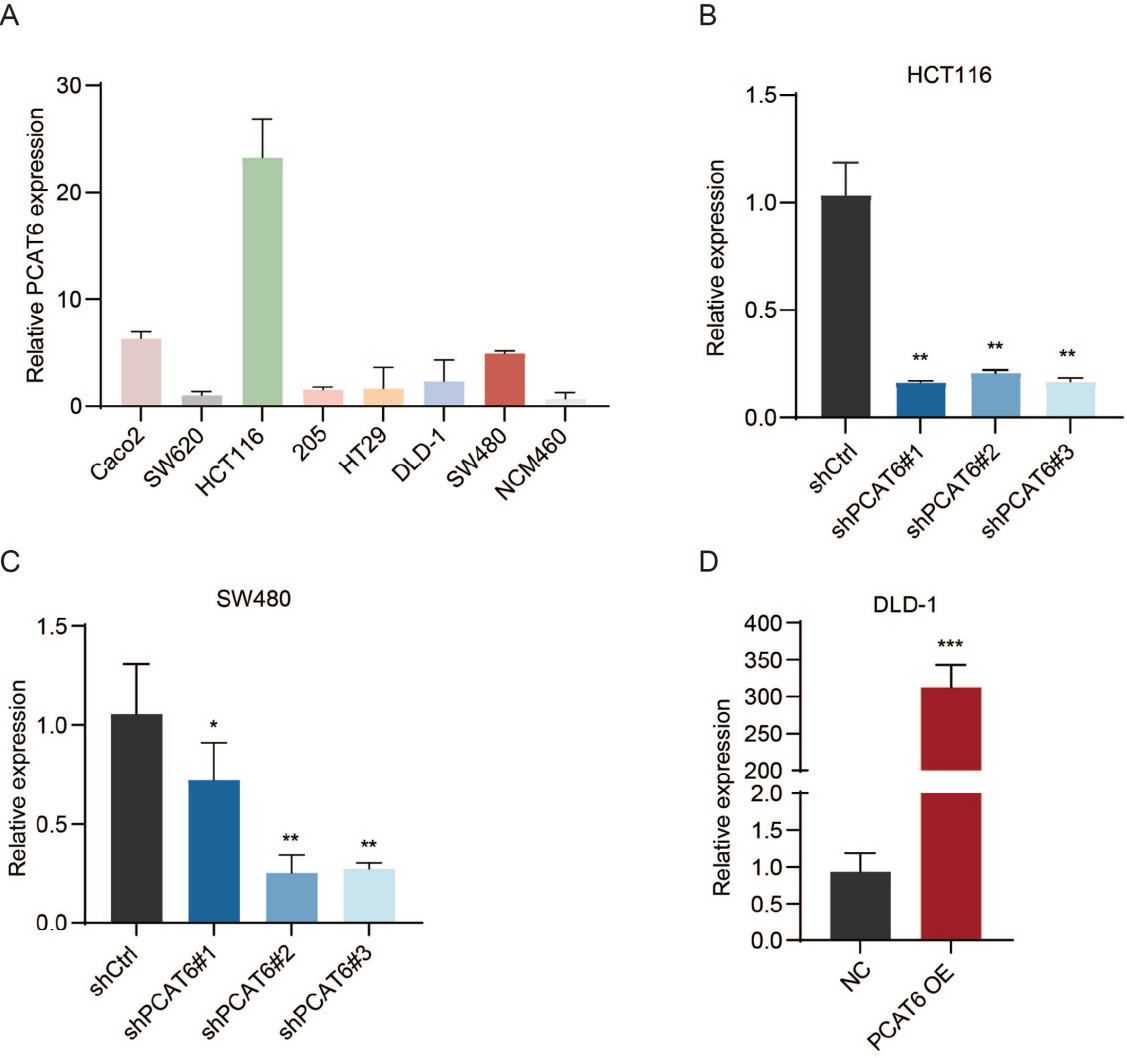
Figure 4 Construction of stably PCAT6-transfected cells (A) RT-qPCR analysis of relative PCAT6 expression in different CRC cells. (B‒D) RT-qPCR was used to confirm PCAT6 knockdown and overexpression efficiencies. *P<0.05, **P<0.01, ***P<0.001.

To further elucidate the molecular mechanism by which PCAT6 influences CRC prognosis, we analyzed PCAT6 expression in seven human CRC cell lines and observed significant differences in the expression levels of PCAT6 ( Figure 4A). We then transfected HCT116 and SW480 cells, which exhibit high PCAT6 expression, with PCAT6-shRNA lentivirus and DLD-1 cells, which exhibit low PCAT6 expression, with a PCAT6-overexpression lentivirus ( Figure 4B‒D). shRNA#2 and shRNA#3 were selected for subsequent RT-qPCR analysis.

**Figure 5:**
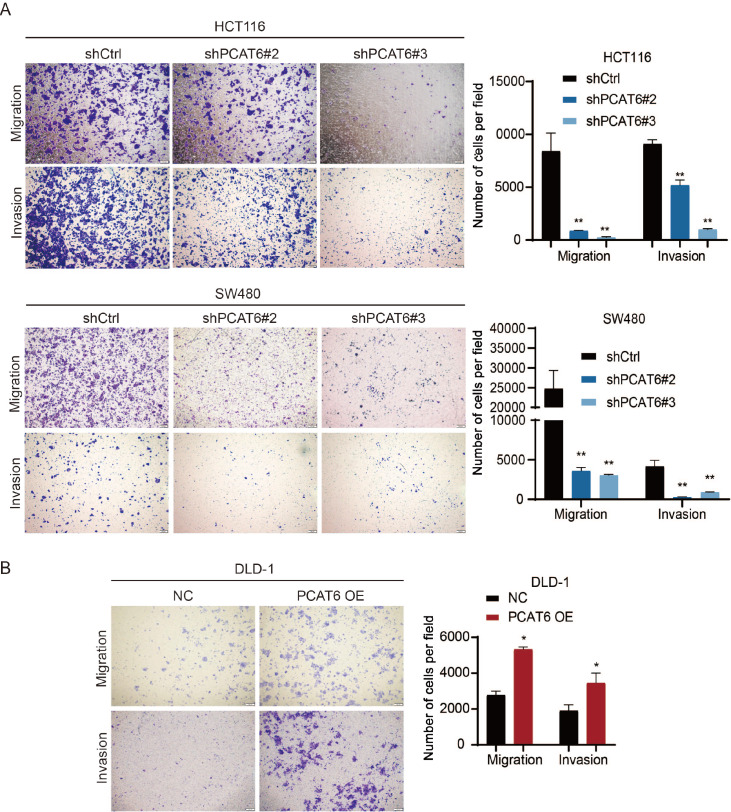
Figure 5 PCAT6 promotes colorectal cancer cell migration and invasion *in vitro* (A,B) The effects of PCAT6 knockdown or overexpression on cell migration and invasion were assessed by transwell assays. Scale bar: 200 μm. *P<0.05, **P<0.01.

**Figure 6:**
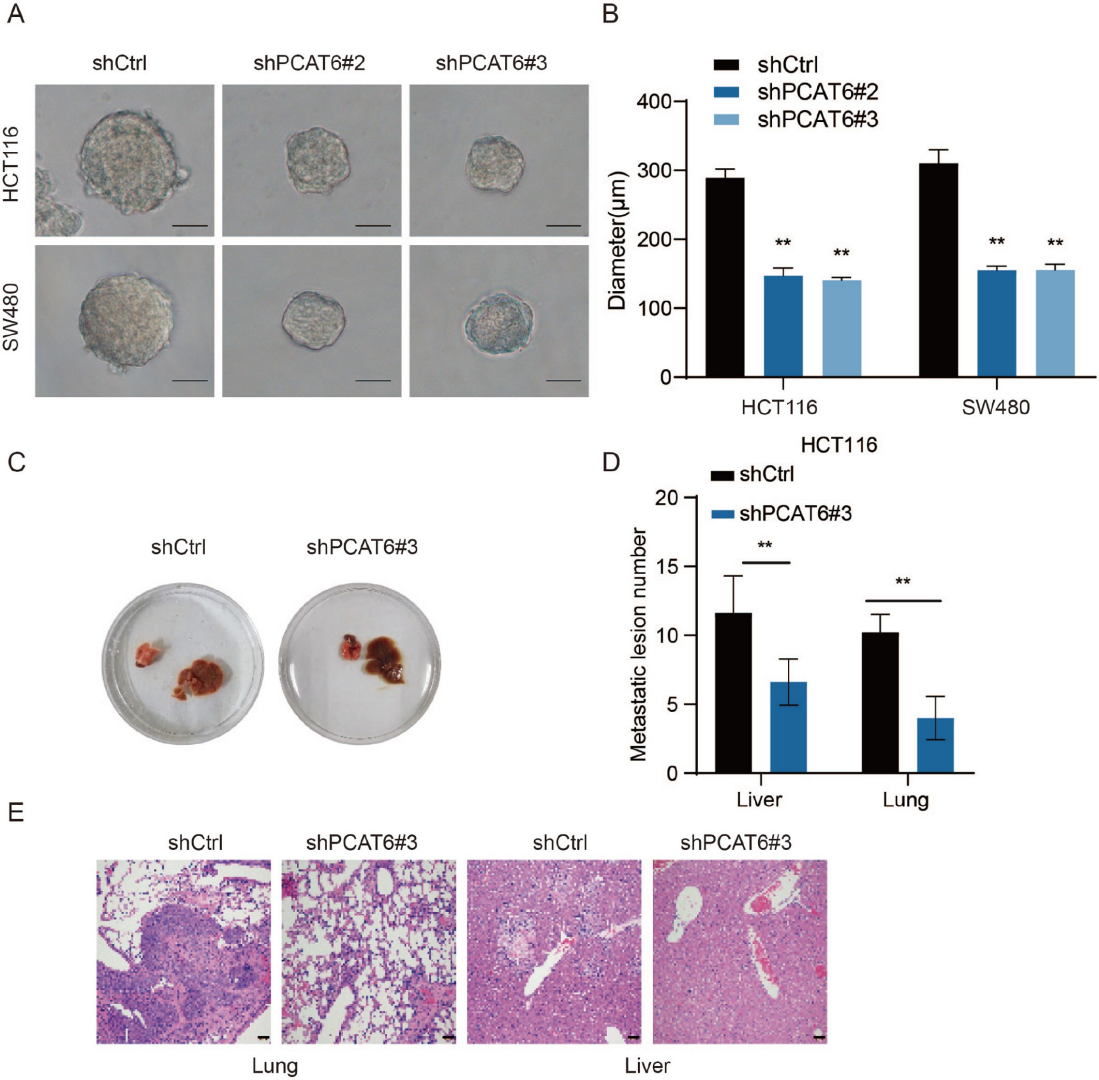
Figure 6 PCAT6 promotes colorectal cancer stemness *in vitro* and metastasis *in vivo* (A,B) The effects of PCAT6 knockdown on sphere-forming ability were detected by sphere-forming assays, and the diameter of spheres was measured. Scale bar: 100 μm. (C‒E) The effects of PCAT6 knockdown on lung and liver metastasis were investigated in vivo, and the number of metastases was examined by hematoxylin and eosin (H&E) staining. Scale bar: 50 μm. *P<0.05, **P<0.01.

The prognosis of patients diagnosed with mCRC has improved but remains suboptimal. Therefore, we investigated the function of PCAT6 in metastasis. We found that *PCAT6* knockdown significantly inhibited the migration and invasion abilities of HCT116 and SW480 cells ( Figure 5A), and PCAT6 overexpression markedly enhanced these abilities in DLD-1 cells, as determined via Transwell assays ( Figure 5B). Notably, Knockdown of *PCAT6* suppressed the sphere-forming ability of CRC cells ( Figure 6A,B). Moreover, in our *in vivo* mouse model, *PCAT6* knockdown inhibited CRC cell liver and lung metastasis ( Figure 6C‒E). These findings indicated that PCAT6 plays a significant role in promoting CRC metastasis.

### *PCAT6* knockdown suppresses the EMT and stemness of CRC cells

**Figure 7:**
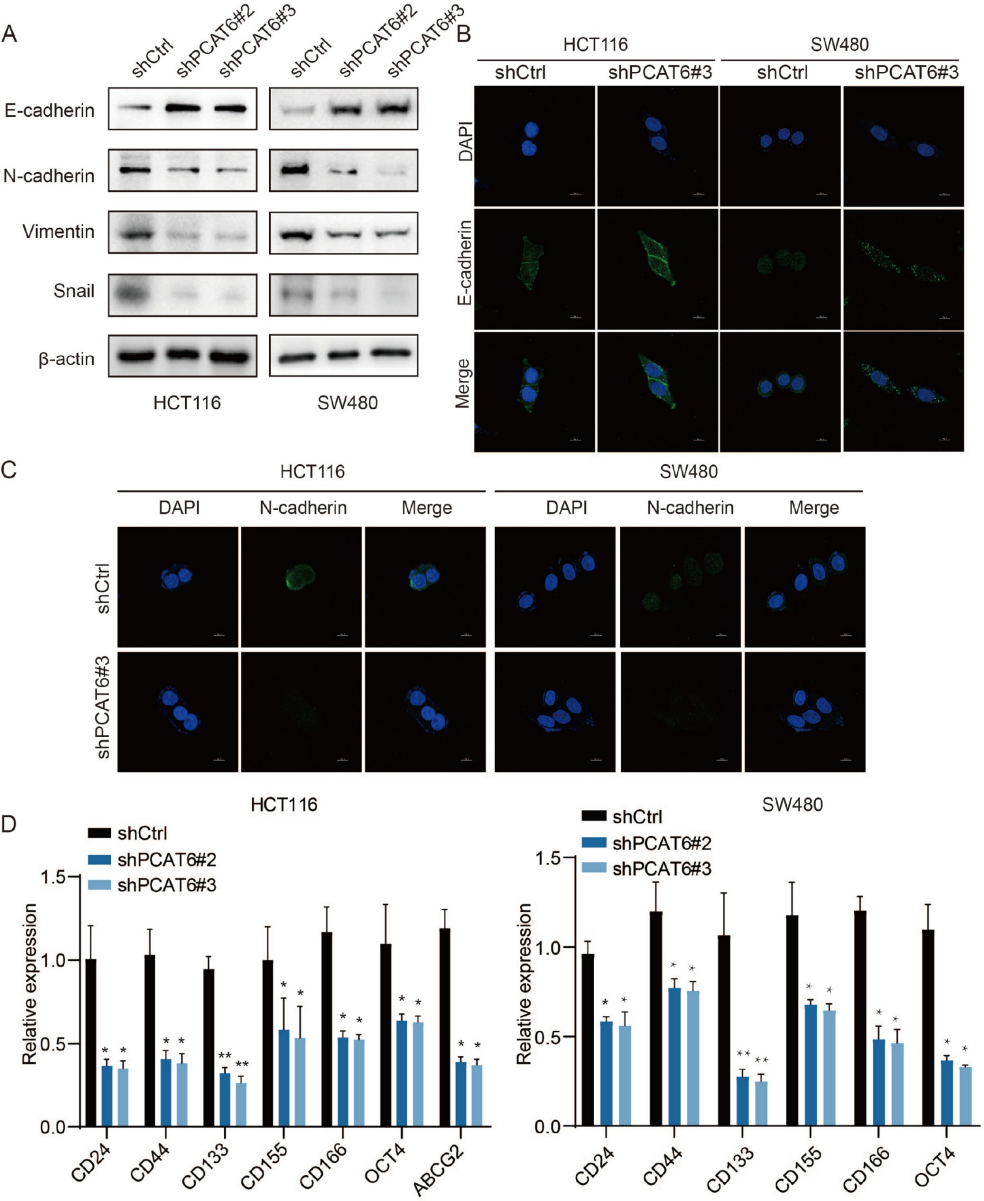
Figure 7 Downregulation of PCAT6 expression suppresses EMT and stemness in colorectal cancer cells (A) Western blot analysis of the relative protein expressions of EMT markers (E-cadherin, N-cadherin, vimentin and Snail). (B,C) The expressions and location of E-cadherin and N-cadherin were analyzed by immunofluorescence staining. Scale bar: 10 μm. (D) RT-qPCR analysis of the relative gene expressions of stemness markers (CD24, CD44, CD133, CD155, CD166, OCT4, and ABCG2). *P<0.05, **P<0.01, ***P<0.001.

EMT and stemness are influential factors in the process of metastasis. Therefore, we further explored the role of PCAT6 in the EMT and stemness of CRC cells. Western blot analysis revealed that E-cadherin expression was greater in HCT116-shPCAT6 and SW480-shPCAT6 cells than in control cells ( Figure 7A). Moreover, N-cadherin, Snail, and vimentin expression levels were lower in HCT116-shPCAT6 and SW480-shPCAT6 cells than those in HCT116 shCtrl and SW480 shCtrl cells ( Figure 7A). The results of the immunofluorescence analysis of E-cadherin and N-cadherin were consistent with the western blot analysis results ( Figure 7B,C). RT-qPCR revealed that the expressions of several markers, including *CD24*, *CD44*, *CD133*, *CD155*, *CD166*, *OCT4*, and *ABCG2*, which are indicators of cancer stemness, were decreased in HCT116-shPCAT6 and SW480-shPCAT6 cells ( Figure 7D). These results indicate that *PCAT6* knockdown inhibits EMT and stemness in CRC cells.

## Discussion

LncRNAs are a class of transcripts exceeding 200 nucleotides in length that are characterized by minimal or no protein-coding potential [ 18‒ 20]. LncRNAs were dismissed ″garbage products″ of transcription, but in the past two decades, their involvement in cell proliferation [ 21, 22], apoptosis [ 23‒ 25], metastasis [ 26, 27], and differentiation [28] has been revealed. Furthermore, lncRNAs regulate tumorigenesis and disease progression at both the transcriptional and posttranscriptional levels [29]. Thus far, differences in the lncRNA expression profiles between early and advanced (with lymph node or distant metastasis) CRC, the potential prognostic value of differentially expressed lncRNAs, and their roles in CRC metastasis remain unclear. In this study, we collected TCGA CRC datasets, analyzed lncRNA expression profiles, screened differentially expressed lncRNAs in cancer tissues, and identified 171 highly expressed lncRNAs in stage III and IV tumors. Furthermore, we showed that PCAT6 expression is an important indicator of poor prognosis in CRC patients. Our comprehensive analyses highlighted PCAT6 as a prognostic factor implicated in CRC metastasis.

PCAT6 was first discovered in 2013 through a comprehensive genomic analysis of different cancer tissues. PCAT6 is actively involved in the pathogenesis of several other cancer types, including prostate cancer [10]; it is overexpressed in almost all types of tumor tissues, and Kaplan-Meier analysis of various tumors revealed that PCAT6 overexpression is weakly correlated with patient survival. Previous univariate/multivariate Cox regression analysis confirmed that PCAT6 expression influences the prognosis of patients with CRC, cervical cancer, liver cancer, and osteosarcoma [10]. According to a meta-analysis involving 937 patients and 8 cancer types, high PCAT6 expression was significantly negatively correlated with overall, progression-free, and disease-free survival, as well as TNM stage and metastasis [30]. In the present study, we analyzed the TCGA CRC dataset and found that the prognosis of patients with CRC exhibiting high PCAT6 expression was poor. Furthermore, PCAT6 was highly expressed in tumors with an invasion depth beyond the deep muscle layer and lymph node metastasis. These results suggested that PCAT6 is an independent factor affecting OS in patients with CRC.

Here we collected 202 CRC tissue samples and performed RNA extraction. PCAT6 expression was verified using RT-qPCR. In the tissue samples from tumors with invasion depths beyond the subserosa and distant metastasis, the OS time of patients with PCAT6-high tumors was significantly shorter. The present results indicated that high PCAT6 expression is suggestive of poor prognosis in CRC patients, highlighting its potential as a prognostic biomarker. Moreover, compared with mRNAs, lncRNAs exhibit good stability and a long half-life, adding to the potential of PCAT6 as a CRC biomarker [31] and warranting further research.

Currently, research has demonstrated the presence of PCAT6 in the plasma of individuals with lung cancer, indicating its inherent stability. ROC curves constructed using plasma PCAT6 levels in patients with lung adenocarcinoma and lung squamous cell carcinoma yielded areas under the curve (AUC) of 0.9213 (sensitivity 87.67%; specificity 97.44%) and 0.9583 (sensitivity 94.12%; specificity 100%) [32], respectively. These AUC values, closely approximating 1, indicated the high sensitivity and specificity of PCAT6 for prognostic prediction. Furthermore, negative correlations between PCAT6 level and OS have been observed in patients with CRC [16], gastric cancer [15], lung cancer [ 33‒ 35], and pancreatic cancer [36]. Conversely, individuals with elevated PCAT6 level in bladder cancer, osteosarcoma, and ovarian cancer patients exhibited shorter OS and progression-free survival (PFS) time. This evidence supports the potential use of PCAT6 as a prognostic biomarker and suggests its applicability for future prognosis detection.

In addition, to gain further insight into the molecular mechanism through which PCAT6 affects CRC prognosis, we established cell lines with stable PCAT6 overexpression or downregulation. Our *in vitro* functional studies showed that PCAT6 significantly promoted the invasion and migration abilities of CRC cells, in addition to promoting EMT. In addition, PCAT6 downregulation suppressed the sphere-forming ability and expressions of stemness markers in CRC cells. Our *in vivo* study using a nude mouse metastasis model showed that CRC cell lines with downregulated PCAT6 expression exhibited reduced metastatic potential. These results suggest that PCAT6 is an lncRNA with extensive cancer-promoting capacity. Moreover, as a regulator of CRC malignancy, PCAT6 holds great potential as a predictive marker for disease recurrence and metastasis.

Huang *et al*. [16] reported that PCAT6 could inhibit apoptosis, and Wu *et al*. [13] reported that PCAT6 plays an important role in regulating chemoresistance in colorectal cancer. However, there is no report on the impact of PCAT6 on the metastasis of colorectal cancer *in vitro* or *in vivo*. Our study revealed the significant role of PCAT6 in the progression of colorectal cancer and elucidated the specific mechanism by which PCAT6 promotes metastasis in colorectal cancer. Our study identified PCAT6 as a potential biomarker for the recurrence and metastasis of colorectal cancer.
